# The enigma of Sir William Robert Wills Wilde (1815–1876)

**DOI:** 10.1177/09677720211046588

**Published:** 2021-10-21

**Authors:** Patrick Boland, Sean P Hughes

**Affiliations:** 1 5803 Memorial Sloan Kettering Cancer Center, New York, New York, United States; 2 4615 Imperial College London, London, UK

**Keywords:** William Wilde, otology, ophthalmology, Irish Census 1841 and 1851, Irish Famine.

## Abstract

William Wilde, father of Oscar Wilde, made a significant contribution to ophthalmology and otology. Qualified as a surgeon. educated in statistics and showing sympathy for the Irish population, Wilde was appointed a Commissioner for the 1851 Census, which covered the time of the Irish Famine (1845–1852). Wilde, steeped in Irish mythology, used his knowledge to develop a close rapport with the Irish peasantry. However, his life was a paradox; he supported the British Government's approach to the Famine and at the same time he showed humanity to the Irish peasantry. In his personal life he was implicated in an abortive libel case involving a young female patient who had accused him of rape. Wilde lived as though he had two separate lives*: on the one hand* the successful surgeon, famine Commissioner and cataloguer of Irish antiquities, and the other a countryman and disciple of Irish mythology. Wilde was highly preceptive especially in his views on the recording of medical data and outcomes in clinical practice. We argue that Wilde was *probably* unmatched in the variety of his talents but was also perplexing in the various actions he took during his life and that indeed Wilde was an enigma.

## Introduction

Many of William Wilde's achievements have been over-shadowed by the success and notoriety of his son, Oscar (1854–1900). However, although William Wilde was an innovator in the field of medicine and surgery, historians hesitate to rank him as a true academic physician scientist.^1^ By today's standards Wilde's education was somewhat unconventional. He did not attend university though he understood the importance of such institutions. As a precocious 23-year-old he criticized Trinity College Dublin for not investing in studies including natural sciences, philology, Semitic languages, and Oriental studies.^2^ Wilde's encyclopedic knowledge of subjects ranging from medicine to natural sciences, art, archeology, Celtic folklore, literature, and statistics singles him out as a man with an avaricious appetite for knowledge enhanced by travel and interactions with the intellectuals of his time.^3^

In this paper, we seek to evaluate the influence of his background and education on his medical, sociopolitical, and artistic achievements. We argue that, while his background and intellectual gifts helped make him ahead of his time, it also resulted in a complex conflicted personality creating an enigma. We also suggest that, despite major upheavals in his life, Wilde was a sympathetic doctor and a kind, committed humanitarian.

## Early years

William Wilde grew up in rural County Roscommon where he frequently accompanied his father Dr Thomas Wilde (1760–1838) on his daily rounds. Apart from influencing his choice of career, these childhood experiences offered William the opportunity to become acquainted with the rural poor, including their ideologies, diet and living conditions. He befriended many and enjoyed listening to their tales of witches, ghosts, saints, and fairies, resulting in a life-long interest in Celtic folklore.^4^ In their journeys, the Wildes would have encountered the underlying agrarian agitation in these deprived, dispossessed people which frequently exploded into rebellion by organized militant groups including the “*Ribbonmen*”, a 19th-century movement of poor Catholics in Ireland. These encounters and rural experiences armed William with valuable insights, setting him apart from his urban middle-class Dublin colleagues.^5^

William Wilde's earliest interests in education, archaeology, and natural sciences were provided by private tutorials, given to him by a retired monk, the Lord Abbott Cong, Father Patrick Prendergast (1741–1829).^6^ This elderly Augustinian cleric, a fluent Irish speaker, lived on the lands owned by his mother's family. At that time Father Patrick had in his possession priceless antiquities which are now to be seen in Dublin's National Museum. Wilde received his early formal education at the Banagher Royal School, in County Offaly, which had been given its royal charter by Charles I. The school was finally established in 1806 and in Wilde's time the Master was the Reverend Dr Alan Bell (1789–1839) a graduate of Trinity College Dublin and Glasgow University. There were 30 to 35 pupil's half of whom were boarders and half day boys. The pupils were the sons of clergymen of the Established Church, of army officers, doctors and other children of the wealthier middle classes of Connaught. The curriculum included Latin and Greek, French, Hebrew and English, algebra, Euclid (geometry), arithmetic (Goughs (practical arithmetic 1812)), geography, and history.^7^ Later Wilde attended at the Elphin Diocesan School, Co Roscommon, which had been founded in 1685 by Doctor John Hodson (1604–1686) Anglican Bishop of Elphin. When Wilde attended the school, the Master was the Reverend William Smith (1801–1841), who was the curate of Elphin and the Vicar General of the Diocese. The curriculum at the Elphin school also included Euclid, algebra, and arithmetic as well as Latin and Greek along with English and bookkeeping, with the pupils aiming for entrance to Trinity College Dublin. Interestingly, at the Elphin school in 1826 there were 19 pupils in attendance of whom 10 were of the Established Church and nine were Roman Catholics and of the total number of 19, 11 were educated for free.^8^ It is therefore clear that William Wilde had an all-round education, learning Irish and about the antiquities from Father Patrick and the classics and mathematics along with history and English from the two schools, which must have laid a sound basis for his future scholarship.

## Medical training

At the age of 17 years, Wilde enrolled as a medical student at Dublin's Park Street Medical School and Dr Steevens’ Hospital where he was apprenticed to the surgeon Abraham Colles (1773–1843) and was taught by the physicians Robert Graves (1793–1853) and William Stokes (1804–1877). By all accounts he was an exceptional student, submitting a prize-winning paper on spina bifida while on his obstetric rotation at the Rotunda Hospital.^9^ In 1837 Wilde was licensed to practice surgery by the Royal College of Surgeons in Ireland and shortly afterwards travelled abroad, chaperoning a wealthy patient of Dr Graves on a Mediterranean cruise. After leaving the patient Wilde spent several months in Egypt which had a defining influence on his life. His interests in botany, zoology and, more significantly, archaeology were stimulated, and he recorded his European travels and explorations in a two-volume book.^10^ Wilde's curiosity in ophthalmology was aroused when in Alexandria and Cairo he encountered the effects of trachoma, a Chlamydial bacterial eye infection that led to partial or complete blindness.

Following his travels to Egypt, to gain further experience in diseases of the eye, Wilde went to London in 1840, spending six months at Moorfield's Eye Hospital. With the help of letters of introduction from the author Maria Edgeworth (1768–1849), he quickly accumulated a group of influential friends including Sir James Clark, (1788–1870), Queen Victoria's physician. Clark, like Wilde, had written a book on his European travels^11^ and had treated the poet John Keats (1795–1821) during Keats’ illness in Rome.^12^ Through Clark, Wilde was introduced to William Farr (1807–1883), epidemiologist and one of the founders of medical statistics.

Intent on furthering his knowledge of ophthalmology and to gain experience in diseases of the ear, in 1840 Wilde travelled to Vienna where he enrolled as an observer at the Allgemeines Krankenhaus.^13^ He made full use of his time in Vienna and recorded his experiences in a book, *Austria: Its Literary, Scientific and Medical Institutions*,^14^ in which, using the new statistical skills he had acquired, Wilde analyzed the Austrian Government's medical, social, and educational polices. Emer O'Sullivan writes in *The Fall of the House of Wilde* (2016) that Wilde was “an intellectual voicing his opinion in Austria's Civil Rights as well as charging the Church and Monarchy with interference in scientific inquiry.”^15^ In Vienna, Wilde demonstrated his qualities of clinical observation when he noted the extremely high rate of puerperal sepsis in the obstetric wards. Wilde believed that the Austrian doctors did not consider puerperal fever infectious, writing “I myself have seen a newly-delivered woman placed in a bed scarcely yet cold, in which a death from puerperal fever had taken place two hours before!”^16^ He was especially critical of the Viennese doctors, castigating them for not taking such elementary precautions, including cleansing, fumigating, whitewashing, and closing infected wards, when managing patients where there was puerperal sepsis.^17^

Some years later, in 1847, Ignaz Semmelweis (1818–1865), who became a life-long friend of Wilde and who also worked at Allgemeines Krankenhaus, took this further. Semmelweis noticed there was an association between contaminated tissue, transported from the autopsy room to the labor ward and borne on the hands of medical students and doctors, and puerperal sepsis.^18^ Semmelweis introduced hand washing with chlorinated lime water dramatically reducing the incidence of puerperal sepsis. These methods were similar to those that Wilde's made seven years earlier when as a medical student he used comparable protocols following the death, from cholera, of a patient he had nursed in Galway.^19^ Both Wilde and Semmelweis's observations were made 20 years before Joseph Lister's (1827–1912) seminal publication on antiseptics in surgery in 1867.^20^ In 1840–1841 Wilde travelled further in Europe, visiting surgical centres and meeting with intellectuals and royalty, showing his ability to be equally at ease with European aristocrats and Irish peasants. In Berlin, he studied with the pioneering plastic surgeon Johann Friedrich Dieffenbach (1792–1847). Later in 1841 he returned to Dublin where he established his first clinic at St Mark's Hospital, dedicated to the treatment of diseases of both the eye and the ear, and largely for poor patients. The hospital had 20 public and three private inpatient beds and became so successful that in 1850 he purchased the recently vacated Park Street Medical School, where he had been a medical student, and transferred St Mark's to that site. This hospital became the premier eye infirmary and one of the only hospitals in the British Isles specializing in diseases of the ear.^21^ Wilde attracted patients to the clinic from all over Ireland, along with students from Europe and North America.

One of Wilde's significant medical contributions was the introduction of meticulous documentation involving the demographics, diagnosis, treatment of patients and, most important, outcomes. In his publications Wilde continued to emphasize the necessity for documentation as well as long-term follow up. In *Practical Observations on Aural Surgery,* he wrote.

statistical tables and calculations are really valuable only when we can rely upon the original investigations from which they were deduced; if the materials have been loosely collected, or for any special purpose, or support any preconceived theory, such circumstances naturally influence the value to be set upon all subsequent arrangements, no matter how ingenious.^22^

This observation remains relevant today, as data input into an analysis has to be accurate or else it has no value and preconceived ideas of the outcome leads to bias.

## Otology and Ophthalmology in Dublin

In 1846, Wilde was appointed editor of the *Dublin Journal of Medical Science,* which enhanced both Dublin's medical reputation and promoted Wilde's career. In the journal, Wilde reported on progress in ophthalmology, citing articles published in the previous 12 months from both home and around the world, based on their scientific qualities.^23^

In *Wilde's Worlds* (2016), the historian James McGeachie states that the medicine practiced by Wilde and his colleagues was not by today's standards truly scientific.^24^ However, the 1830s were the turning point in the importance of the microscope in studying organs and tissues of the body. Microscopy was being developed particularly in Europe and many students from Britain travelled to Europe, notably to Paris, to acquire expertise in this new technology. In Edinburgh in the 1830′s Allen Thompson (1809–1884) was probably the first person to introduce microscopy to medical student teaching^25^ and John Goodsir (1814–1867) conservator of the Museum of the Royal College of Surgeons of Edinburgh and later Professor of Anatomy at Edinburgh University, developed his theory about the nature and structure of cellular life and organization based on microscopic evaluation of tissues.^26^ Later, this new science of microscopy became widely available for the study of gross pathological features following the seminal work of Rudolf Virchow (1821–1902) in 1858.^27^ Hence, the scientific revolution in medicine was on its way and Wilde recognized the importance of understanding anatomy and pathology, particularly of the organs of hearing. He collaborated with the London otologist Joseph Toynbee (1815–1866), who had dedicated his life to the study of the anatomy and pathology of the ear, and it is apparent that Toynbee's talents as a scientist complemented the clinical expertise of Wilde.^28^ Wilde's understanding of the role of a physician are to be found in his book *Practical, Observations on Aural Surgery* in which he wrote “The practitioner of aural surgery ought to be a well-educated surgeon or physician who applies the well-recognized principles of medicine to the organ of hearing.”^29^ Wilde paid great attention to the classification and tabulation of diseases of the eye and ear, as well as reporting the outcomes of these conditions. Fifty years later Ernest Armory Codman (1869–1940), a surgeon in Boston, Massachusetts, USA, devoted much of his life to advocating the accountability of hospitals and physicians ‘*The End Result Idea*’ (1918).^30^ Controversial at that time, the concepts of these two pioneers have become the standard practice for hospitals and physicians and surgeons today. Wilde also made substantial contributions to advances in surgical practice by inventing instruments for use in otology and describing a post-auricular incision for draining periosteal mastoid abscesses, Wilde's Incision.^31^ Further evidence of his progressive scientific views was his early adaption of chloroform anaesthesia and the use of atropine eyedrops in the management of inflammatory diseases of the eye.^32^ In 1850 he championed the empirical use of cod liver oil, a dietary supplement rich in vitamins A and D, in the management of xerophthalmia, a condition characterized by progressive dryness of the eye, which can lead to blindness and is now known to be due to vitamin A deficiency.^33^

Wilde's scientific approach to the practice of surgery could however be seen to be at odds with his strongly held beliefs in mythology and folklore. It would appear though that he was at ease with these disparate topics, dealing with these conflicting positions entirely separately.

## The Irish census

The historian Peter Froggatt asserted that the appointment of Wilde to the Irish Census Board was inspirational.^34^ Wilde's medical qualifications, statistical knowledge, familiarity with the Irish language and folklore along with publications on social issues all made him an ideal choice. As a young doctor, Wilde also welcomed the added income. He was first appointed to the Commission as a medical advisor and compiler of the causes of death from the 1841 Census. The Commissioner at that time, Sir Thomas Lorcan (1801–1879), was impressed by Wilde's work and appointed him Assistant Commissioner for the 1851 Census, a position he held for the rest of his life.

Wilde approached the Herculean task of conducting the 1851 Census, which covered the years of the Great Famine, with his usual vigor, presenting a detailed completed report in 1856. This is the only official audit of the Great Famine carried out by the British State and Froggatt wrote that it was one of the greatest national Censuses ever produced, including details of the incidence of impaired hearing and sight as well as mental disabilities, which was unique at the time.^35^ In the report, Wilde also recorded the incidence of blindness on 31 March 1851 in both adults and children as one person in 864, later using this information to draw the attention of the authorities to provide more facilities for the care of blind patients. Also, at Wilde's insistence the 1851 Census included the inhabitants of both the workhouses and hospitals who had been ignored in previous censuses.^36^

However, despite Wilde's best organization and analytical endeavors, the Census Reports were flawed. They relied primarily on the honesty and memory of an exhausted and sometimes hostile population stretching back over 10 years during which time many families had died or emigrated. Wilde appears to have been aware of these shortcomings, now known as bias, but was not deterred by them. He used his rural background knowledge to interpret and enhance the value of the information he collected. Also, as editor of the *Dublin Quarterly Journal*, he circulated questionnaires to local doctors to improve the accuracy of the data.^37^

However, William MacArthur, the historian, in 1956 commented that many historians placed little credence on the usefulness of Wilde's tables.^38^ Later though in 2002, the economic historians Joel Mokyr and Cormac O’Grada applied mathematical modeling to Wilde's data and concluded that the Census reflected the conditions of that time.^39^ In *Power and Popular Culture* (2010),^40^ Peter Gray discussed the accuracy of Wilde's figures and noted that, before his publication of the tables of deaths in the General Census Report in 1856, there was wide variation in the mortality reported by the press. *The Times* of London, for example, estimated that there were about 20,000 deaths from the famine, while the nationalist Irish *Freeman*'s *Journal* put the figure at 4.75 million. Wilde in the final Census reported a decline in the general population of 2.5 million with deaths accounting for one million, and emigration accounting for much of the remainder. These estimates were accepted later by both *The Times*^41^ and the *Freeman's Journal.*^42^ The latter publication admired Wilde's work but was highly critical of his failure to lay blame for the catastrophe on the British Government. Indeed, at that time the liberal Irish press, notably *The Nation*, championed the writings of the radical John Mitchel (1815–1875) who wrote “The Almighty, indeed, sent the potato blight, but the English created the Famine”.^43^ Wilde though in his Census Report, had defended the policies of both the Dublin and the London Governments and, in *The Food of the Irish* published in 1854, stated that the Famine had cleared the way for the modernization of Irish society and its diet, especially the potato that supported it. Wilde concluded that the substantial reduction in the population produced by the famine led to improvement in agriculture, living conditions and literacy of the Irish poor. He strongly criticized the dependency of the Irish peasantry on the potato because when it was plentiful it made them lazy and nonproductive, which fuelled agitation. Wilde asked, “Have we lost a man too many? The best friends of Ireland and her people would say not,”^44^ revealing a remarkable lack of empathy toward the Irish peasantry. This paradox in Wilde's personality is perpetuated throughout his life.

## Highs and lows

In the 1850s and early 1860s, William Wilde was at the zenith of his fame. He was an internationally recognized doctor and had written successful books on surgery, antiquities, and folklore.

In March 1857 he undertook the massive project of cataloguing, describing and illustrating some 10,000 antiquities in the museum of the Royal Irish Academy. He successfully completed this task in four months, which a committee of experts had failed to accomplish in four years. The publication of this catalogue for which he received the Academy's highest award, the Cunningham Medal, marked the end of the greatest productive period of Wilde's life.^45^ Wilde was also the author of a National Census Report unequalled internationally, and he was awarded a knighthood of the Order of St Patrick for this work in 1864. Wilde himself concludes that all this work had been done at a risk to his life.^46^ and Froggatt posits that it was the beginning of his mental and physical decline.^47^

Despite this success Wilde displayed a somewhat reckless side to his character. He had several illegitimate children, and it was common knowledge that he was a philanderer.^48^

Furthermore, in the mid-1864 his career suffered a severe blow which could be attributed to irresponsible behaviour. He had developed an unprofessional relationship with a young female patient named Mary Travers who became so demanding that Wilde tried to end the relationship. Determined to ruin Wilde, Mary Travers circulated leaflets suggesting he had raped her while she was under the influence of chloroform. Lady Jane Wilde (1821–1986), William's wife and writer, known as Speranza, was incensed by these accusations and wrote to Mary's father protesting at these allegations. Mary, though, read the letter and started proceedings to sue Lady Jane for libel, thereby implicating William Wilde. The court found in favour of Mary Travers and ruled that Wilde was not without fault, but only awarded damages of one farthing. However, Wilde was left with legal fees of £2000 on behalf of Lady Jane.^49^ Though *The Lancet* in London, *Saunders's Newsletter* in Dublin, and most of the medical fraternity, supported him, after the court case Wilson notes that Wilde lost his enthusiasm for surgery and that his clinical practice suffered irrevocably following the trial.^50^ Following the court case Wilde departed Dublin for Moytura near the village of Cong in County Mayo, leaving his practice in the care of Henry Wilson who was widely thought to be Wilde's illegitimate son.^51^

In *The Parents of Oscar Wilde* (1967) Terence de Vere White wrote that even before the trial Wilde “had taken to escaping to Cong whenever he could”, indicating that his medical interests were already waning.^52^ Richard Ellman, Oscar Wilde's biographer, suggests that Wilde showed his indifference to the outcome of the lawsuit “by writing his most cheerful book *Lough Corrib* (1867)*.*”^53^

Unfortunately, Wilde suffered further anguish when in 1867 his daughter Isola, died in her tenth year, most likely from meningoencephalitis, a fatal brain complication of typhus,^54^ and then three years later his two illegitimate daughters died in a fire. Wilde had been suffering from asthma and bronchitis for many years and his general health started to deteriorate significantly in late 1875, although Froggatt suggested that his decline dated back to the mid-1850s.^55^ Wilde died on 19 April 1876 aged 61 years.

## Humanitarian

The literature is replete with reports of Wilde the humanitarian, an example of which is when as a medical student he travelled to Connaught and nursed a terminally ill man suffering from cholera, whom he personally arranged to be buried.^56^ This characteristic was highlighted in an obituary in the *Journal of the Archeological Society* in October 1876 which reported

By the peasantry he was particularly loved and trusted, for he had brought back joy and hope to many households. How gratefully they remembered his professional skill, always so generously given, and how, in the remote country districts, he would often cross moor and mountain at the summons of some poor sufferer, who believed with simple faith that the *Doctor mor* (the great Doctor as they called him) would certainly restore the blessed light of heaven to blind-struck eyes.^57^

Wilde also showed real concern for the health of his medical colleagues, especially those working in the Poor Law Service. As editor of the *Quarterly Journal*, he highlighted the appallingly high mortality among dispensary doctors, who were contracted to give medical care to the poor, by publishing an article by the surgeon James Cusack (1778–1861) and the physician William Stokes.^58^ The article used figures derived from Wilde's Tables of Deaths in the 1841 Census and these data were presented to a Select Committee of the House of Commons. Wilde also published a letter by Robert Graves, which severely criticized the Board of Health for its treatment of dispensary doctors who at that time were dying at an alarming rate.^59^ Wilde, Cusack, and Stokes established a medical relief committee which distributed money to the families of those doctors who had experienced hardship.^60^ Moreover, he demonstrated his generosity by opening St Mark's Hospital largely for poor patients and in 1862 he bequeathed his hospital to the city, for the use of the poor of Ireland, probably the greatest example of his philanthropy.^61^

## Conclusion

William Robert Wilde's life included many major achievements, some of which were influenced by his background and early education. His considerable accomplishments though could not have occurred without an insatiable appetite for knowledge in many diverse spheres of life, combined with an enthusiasm and determination to make advances in the fields of otology and ophthalmology. His insistence on the accurate documentation of clinical data, the use of statistical methods, and the early introduction of the concept of evidence-based medicine, shows him to have been much ahead of his time. Some of Wilde's observations were prescient, including the infectious nature of puerperal fever and the treatment of xerophthalmia. These contributions to medicine rank him as an original physician scientist.

Wilde also demonstrated a keen sense of scholarship from his earliest days, not only in medicine but also in such divergent disciplines as archaeology and mythology. His accomplishments as a Census Commissioner and his classification of the antiquities in the Royal Irish Academy show the breadth of his interests and intellect.

By contrast, it has to be acknowledged however that Wilde's life was full of conflicts and contradictions. On the one hand in his professional career, he was a strong advocate of evidence-based medicine, on the other, in his writing on mythology, he recommended folk remedies.^62^ His reliance on common sense and proven practices in running his hospital contrasts with his beliefs in mythological events such as the Battle of Moytura in 3303 BC.^63^ These apparent paradoxes may be explained by understanding that Wilde's primary vocation was as a scientific physician while his genuine interest in Celticism and folklore was romantic and can best be described as an avocation.

It would also appear that his reports on the Great Famine were surrounded by an internal conflict. For example, in the 1851 Census Wilde defended the Whig Government's management of the disaster, even lending support to Charles Trevelyan's (1807–1886) ‘*The Irish Crisis 1848’* which attributed the catastrophic effects of the famine to Divine intervention.^64^ However, McGeachie notes that while writing the 1851 Census report,

Wilde's authorial voice moved from being a government-appointed exponent of one the key legacies in Ireland of the Enlightenment State – the tabulating of public health – to that of the narrator of a tragedy, the massive human, social and cultural catastrophe of the famine.^65^

However, the fact that he largely absolved the British Government of blame is difficult to understand, coming from a man with firsthand experience of British oppression of the Irish peasants in his youth. Possible explanations for this dichotomy are that he was acting as an insensitive civil servant, unwilling to bite the hand that fed him, an explanation that would suggest a certain weakness of character. This fault may also have accounted for the fact that he did not defend himself in the Mary Travers case, and also that later he avoided discussion with his family about his precarious financial status, which condemned his widow to a life of genteel poverty.^66^ Another possible explanation was that although he genuinely believed what he wrote, he modified his beliefs over the passage of time. In the last speech of his life, delivered in 1874 to the Anthropological Section of the Royal Academy in Belfast, Wilde stated bluntly that Ireland was not defeated by Henry II but by the Great Famine which was made worse by British Government policy.^67^

Wilde's conventional public lifestyle contrasts with his private behaviour such as demonstrated by his illicit affairs. To explain his complexities and eccentric conduct, it has been suggested that Wilde had a form of Asperger's Syndrome, a neurodevelopmental disorder characterized by difficulties in social interaction and nonverbal communication, believed to be common in Irish artists of his time.^68^

Wilson states that the loss of his court case in 1864 was a severe blow from which Wilde's medical career never recovered^69^ although the evidence might suggest otherwise. It would appear that his interest in surgery diminished, possibly as early as 1862 when he handed over the management of St Mark's to a board of trustees and his son, Henry Wilson, alleged to be Oscar's half-brother, took over a large part of Wilde's ophthalmological practice.^70^ Froggatt argued coherently though that Wilde's decline probably began even earlier and could be traced to the physical and psychological exhaustion he suffered after completing the classification of antiquities for the Royal Irish Society in 1857.^71^ It is conceivable that after more than 20 years of stressful medical practice and running a hospital Wilde suffered from a condition now known as “medical burn out.”^72^ Nonetheless, Wilde continued with his non-medical interests, even writing his *Lough Corrib. Its Shores and Islands*, thought by many to be his finest work*,* suggesting that burn out only affected his medical practice.^73^

What is also fascinating is that, in addition to his liberal humanitarian qualities, Wilde was an outspoken advocate for racial equality and supported the principles of crossbreeding. This was at a time when many Europeans were promoting racial purity and white superiority.^74^

In conclusion, we would argue that William Wilde was a thoughtful, kind, humane doctor although his interpretation of the effects of the Irish Famine being of benefit to the Irish people would not sit comfortably with modern opinions. He was a true medical scientist who could empathize with both the medical elite in Dublin's Merrion Square and with the peasants in Moytura.

Perhaps his life is best summed up by LB Somerville-Large (1901–1966) who wrote.

Let us then remember William Wilde: certainly not just as the father of Oscar, but as a great humanitarian, a great doctor, a unique statistician, an investigator in many fields, and also as one who loved to fish in Connemara, to study his country's archaeology, but who loved perhaps best of all the company of his friends.^75^

In reality Wilde was an enigma. He promoted his wide-ranging ideas in articles and books, primarily to make advances in his specialty. He understood statistics and the need for outcome data to be recorded in order to improve the management of patients. He delighted in the study of architecture as well as cataloguing the antiquities. However, he lived an alternative life embedded in mysticism and romanticism and was reckless with his social life and finances. We would suggest however, that without such innovators and Enlightenment figures as Wilde, who are in turn extremely individualistic, single-mindedly pursuing their ideals, the world would be a far poorer place.
